# The origin, type and hydrocarbon generation potential of organic matter in a marine-continental transitional facies shale succession (Qaidam Basin, China)

**DOI:** 10.1038/s41598-018-25051-1

**Published:** 2018-04-26

**Authors:** Guo-Cang Wang, Min-Zhuo Sun, Shu-Fang Gao, Li Tang

**Affiliations:** 1 Key Laboratory of Petroleum Resources, Gansu Province/Key Laboratory of Petroleum Resources Research, Institute of Geology and Geophysics/The Analytical Service center, Research Center of Oil and Gas Resources, Northwest Institute of Eco-environment and Resources, CAS, Lanzhou, 730000 PR China; 2 PetroChina Qinhai Oilfield Research Institute of Exploration & Development, Dunhuang, 736202 PR China

## Abstract

This organic-rich shale was analyzed to determine the type, origin, maturity and depositional environment of the organic matter and to evaluate the hydrocarbon generation potential of the shale. This study is based on geochemical (total carbon content, Rock-Eval pyrolysis and the molecular composition of hydrocarbons) and whole-rock petrographic (maceral composition) analyses. The petrographic analyses show that the shale penetrated by the Chaiye 2 well contains large amounts of vitrinite and sapropelinite and that the organic matter within these rocks is type III and highly mature. The geochemical analyses show that these rocks are characterized by high total organic carbon contents and that the organic matter is derived from a mix of terrestrial and marine sources and highly mature. These geochemical characteristics are consistent with the results of the petrographic analyses. The large amounts of organic matter in the Carboniferous shale succession penetrated by the Chaiye 2 well may be due to good preservation under hypersaline lacustrine and anoxic marine conditions. Consequently, the studied shale possesses very good hydrocarbon generation potential because of the presence of large amounts of highly mature type III organic matter.

## Introduction

Because of the successful commercial exploration and development of shale gas in the USA^1–3^, large investments in the exploration and production of shale gas, an alternative hydrocarbon resource, have been made in China in recent years^4,5^. At the same time, studies have yielded many notable findings regarding depositional environments of marine or terrestrial shales, the formation conditions, accumulation mechanisms and enrichment patterns of shale gas, and the distribution of shale gas reservoirs^6–9^. However, to date, only a few geologists have investigated the geochemical and reservoir characteristics, gas-bearing conditions, shale gas accumulation conditions, and exploration prospects of transitional facies between marine and terrestrial environments^10,11^. The area investigated in this study is located in the Delingha depression of the Qaidam Basin in western China. Despite the relatively numerous oil exploration studies in this area^12–14^, few studies on the organic geochemical characteristics or hydrocarbon generation potential of the marine-terrigenous facies shale have been published^15^. Cao (2016) simply analyzed the type, amount and maturity of the organic matter on the marine-terrigenous facies shale and mainly studied characteristics and factor of shale gas in this area^15^, but origin of the organic matter and hydrocarbon generation potential were not reported. In this study, organic geochemical (especially biomarkers) and petrographic methods were used to examine the marine-terrigenous facies shale found in the Delingha depression of the Qaidam Basin. The objectives of this study were to determine the type, amount, origin and maturity of the organic matter found in these rocks as a function of their depositional environment and to evaluate their hydrocarbon generation potential. A series of analyses, including gas chromatography–mass spectrometry (GC-MS), Rock-Eval pyrolysis, total organic carbon (TOC) evaluation, vitrinite reflectance assessment, and maceral group identification, were performed to meet these goals.

## Geological Background

A total of 36 shale samples were collected from depths between 40 and 1050.8 meters in the Chaiye 2 well, which is located in the Qaidam Basin, China. The northern margin of the Qaidam Basin is one of the key onshore petroliferous blocks within the basin. It is composed of two first-order tectonic units and eleven second-order tectonic units. The first-order tectonic units are the faulted block of the northern margin and the Delingha depression. The faulted block of the northern margin includes eight tectonic subunits, namely, the Tuonan tectonic belt, the Saishiteng sag, the Yuqia-Hongshan sag, the Lenghu tectonic belt, the Yibei sag, the Kunteyi sag, the Eboliang tectonic belt and the Dahonggou swell. The Delingha depression can be divided into three subunits, specifically, the Tuobei tectonic belt, the Tuosuhu sag and the Wulan tectonic belt.

The Delingha depression covers an NW-SE-oriented area of approximately 1.8 × 104 km^2^ and contains a Jurassic intracontinental rift basin superimposed on an early Paleozoic passive continental margin rift basin and a Neopaleozoic back-arc rift basin. The tectonic deformation within the Delingha depression is intense, and local structures are well developed. The development of the Delingha depression was strongly controlled by the tectonic evolution of the Xitie, Oulongbuluke, Aimunike and Qilian Shan orogenic belts; thus, the sag zone between the orogenic belts is the most favorable area for oil and gas exploration.

**Figure 1:**
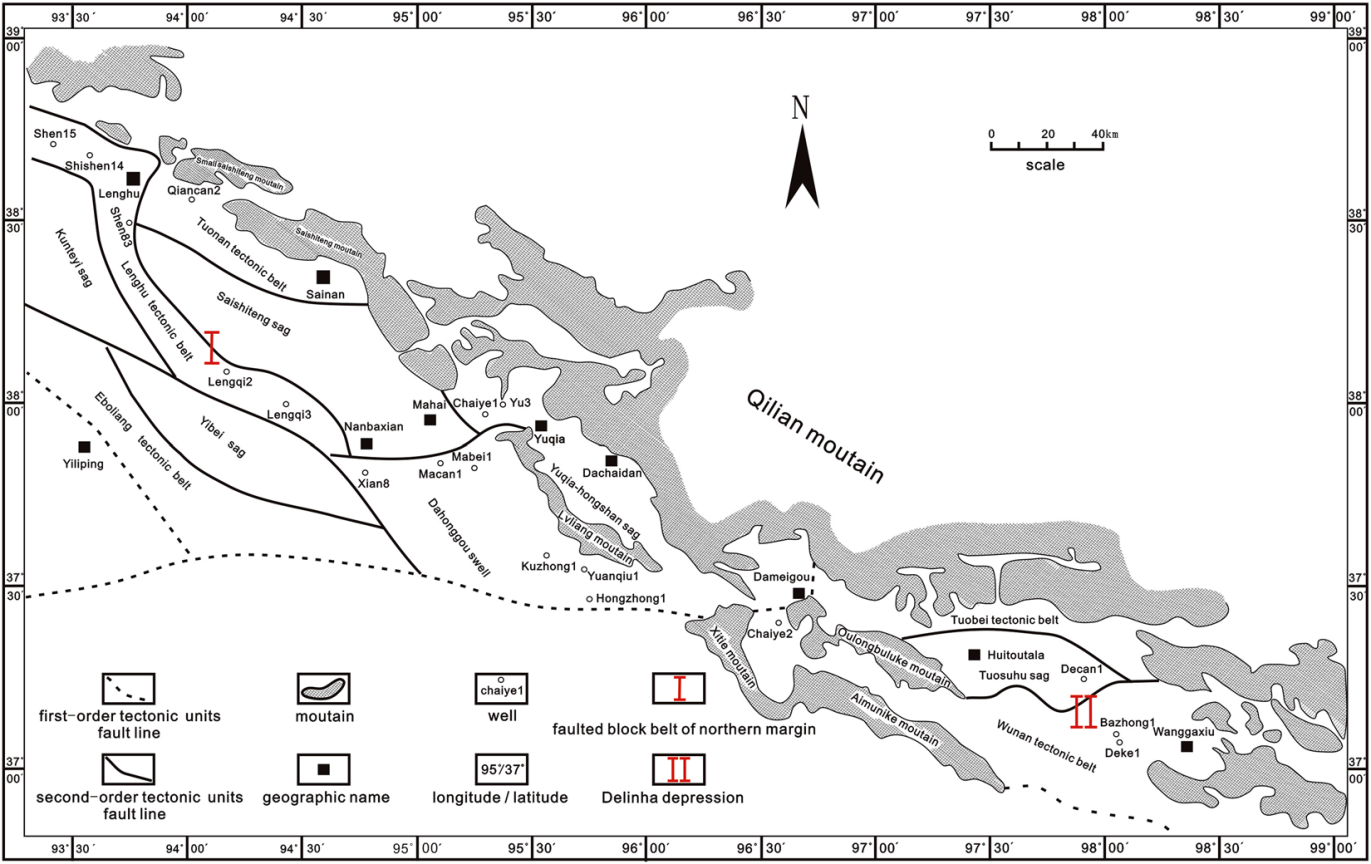
Tectonic units in the Qaidam Basin and the location of the Chaiye 2 well. The maps were created using CorelDRAWGraphicsSuite2017Installer_RW (http://www.coreldraw.com/cn/product/graphic-design-software/trial-thank-you.html?ver=18.0).

The sedimentary sequence within the Delingha depression is well developed and preserved, and it contains Cambrian marine carbonates, Ordovician marine carbonate-detrital rocks, Devonian green and purple detrital rocks, Carboniferous marine-continental transitional sedimentary rocks and Permian shallow-sea and deep-sea marine carbonate and detrital rocks. The Carboniferous strata are composed of the lower Carboniferous Chengqianggou (C1c) and Huitoutala (C1h) formations and the upper Carboniferous Keluke (C2k) and Zhabusagaxiu formations. These strata represent the marine-continental transitional sedimentary facies. The Chaiye 2 well is located within the Wulan tectonic belt and penetrates the upper Carboniferous Keluke formation. This unit is mainly composed of dark shale, mudstone, carbonaceous shale and coal^16,17^ (Fig. 1).

## Results

### TOC and Rock-Eval pyrolysis

To investigate the origin, type and hydrocarbon generation potential of shale, we studied the characteristic of its insoluble organic matter by Rock-Eval to determine the Total Organic Carbon content(TOC), free hydrocarbons (S1), hydrocarbon generative potential (S2), temperature (Tmax) at themaximum of the S2 peak, and production index (PI = S1/(S1 + S2)), hydrogen index (HI = S2/TOC × 100). The Rock-Eval data and the PI and HI values of the samples are listed in Table 1. The TOC contents of the 36 shale samples ranged from 0.29% to 14.10%, and the average TOC content of all samples was 3.75%. The S1 values of the studied shale samples ranged from 0.03 mg HC/g to 1.84 mg HC/g, and the S2 values ranged from 0.14 mg HC/g to 13.46 mg HC/g. The Tmax values of the shale samples ranged from 443 °C to 460 °C, with an average value of 452 °C. The HI values of the shale samples ranged from 10.28 mg HC/g TOC to 102.51 mg HC/g TOC, with an average value of 32.41 mg HC/g TOC. The PI values of the shale samples ranged from 0.09 to 0.32, with an average value of 0.18.

**Table 1:** Main geochemical characteristics of pyrolysis study on shale samples, as determined by Rock-Eval analysis.

Sample	Depth (m)	Lithology	TOC (wt %)	Rock-Eval pyrolysis				
				S1 (mg HC/g)	S2 (mg HC/g)	Tmax (°C)	HI (mg HC/g TOC)	PI
CY1	40	black mudstone	3.77	0.17	0.82	445	21.75	0.17
CY2	100	ashen mudstone	0.71	0.03	0.17	445	23.94	0.15
CY3	150	black mudstone	3.79	0.17	0.76	451	20.05	0.18
CY4	245	black shale	3.83	0.34	3.07	447	80.16	0.10
CY5	332	black shale	2.97	0.07	0.43	444	14.48	0.14
CY6	395	gray-black shale	1.50	0.05	0.21	443	14.00	0.19
CY7	476	black shale	2.06	0.31	0.99	455	48.06	0.24
CY8	514	black carbonaceous shale	7.27	0.21	0.82	445	11.28	0.20
CY9	603	black coal	13.13	1.84	13.46	452	102.51	0.12
CY10	653.63	gray shale	0.72	0.10	0.25	460	34.72	0.29
CY11	654.03	gray shale	0.30	0.03	0.14	443	46.67	0.18
CY12	654.75	black shale	3.39	0.33	0.76	460	22.42	0.30
CY13	655.42	gray shale	0.29	0.08	0.16	460	55.17	0.33
CY14	657.03	black shale	2.67	0.13	0.30	456	11.24	0.30
CY15	657.43	black shale	2.53	0.62	1.39	447	54.94	0.31
CY16	660.73	black carbonaceous shale	6.94	0.70	3.66	455	52.74	0.16
CY17	661.28	black coal	14.10	1.29	11.60	452	82.27	0.10
CY18	840.6	black shale	2.43	0.14	0.44	457	18.11	0.24
CY19	846.1	black shale	2.72	0.15	0.66	448	24.26	0.19
CY20	902.56	gray-black shale	1.18	0.07	0.26	446	22.03	0.21
CY21	911.37	gray-black shale	1.69	0.09	0.35	445	20.71	0.20
CY22	918.07	black carbonaceous shale	8.51	0.50	3.70	448	43.48	0.12
CY23	920.39	black shale	2.06	0.06	0.33	462	16.02	0.15
CY24	933.94	gray-black shale	3.74	0.10	0.43	446	11.50	0.19
CY25	938	black shale	2.53	0.14	0.94	448	37.15	0.13
CY26	954.1	black shale	2.14	0.03	0.22	458	10.28	0.12
CY27	970.3	gray shale	0.89	0.04	0.2	453	22.47	0.17
CY28	988.05	black shale	2.83	0.14	0.74	454	26.15	0.16
CY29	994.15	black limestone	2.31	0.12	0.54	456	23.38	0.18
CY30	997.15	black shale	4.49	0.19	1.19	455	26.50	0.14
CY31	999.2	black shale	2.32	0.12	0.72	458	31.03	0.14
CY32	1019.3	black shale	2.18	0.03	0.23	459	10.55	0.12
CY33	1035.1	black carbonaceous shale	9.28	0.43	3.44	459	37.07	0.11
CY34	1035.3	black shale	3.40	0.14	0.60	451	17.65	0.19
CY35	1048.7	black carbonaceous shale	8.72	0.37	3.79	458	43.46	0.09
CY36	1050.8	gray-black shale	1.54	0.07	0.44	466	28.57	0.14

Total Organic Carbon (TOC) is given in percent. S1 represents free hydrocarbons, S2 represents the hydrocarbon generative potential, Tmax represents the temperature at themaximum of the S2 peak. Production index (PI = S1/[S1 + S2]) and hydrogen index (HI = S2/TOC × 100).

### Vitrinite reflectance and maceral groups

The vitrinite reflectance values (Ro, %) of the samples range from 1.09–1.53%. The Ro values of 80% of the samples fell within the range of 1.3–2.0%, and the Ro values of 20% of the samples fell within the range of 0.7–1.3% (Table 2).

**Table 2:** Maceral groups, the vitrinite reflectance values (Ro) and proximate analysis of the studied shale samples.

Sample	Depth (m)	Lithology	Ro (%)	a (%)	b (%)	c (%)	d (%)	KTI	Organic matter type
CY4	245	black shale	1.09	41.68	Trace	57.82	0.5	−2.2	III
CY7	476	black shale	1.20	39.06	Trace	60.12	0.82	−6.9	III
CY9	603	black coal	1.17	34.49	Trace	64.31	1.2	−14.9	III
CY13	655.42	gray shale	1.30	31.12	Trace	67.98	0.9	−20.8	III
CY17	661.28	black coal	1.19	21.21	Trace	76.84	1.95	−38.4	III
CY18	840.6	black shale	1.38	22.26	Trace	76.14	1.6	−36.4	III
CY20	902.56	gray-black shale	1.49	36.18	Trace	62.72	1.1	−12.0	III
CY21	911.37	gray-black shale	1.46	40.15	Trace	59.29	0.56	−4.9	III
CY22	918.07	black carbonaceous shale	1.37	38.64	Trace	60.96	0.4	−7.5	III
CY24	933.94	gray-black shale	1.46	40.83	Trace	58.27	0.9	−3.8	III
CY25	938	black shale	1.43	29.19	Trace	69.01	1.8	−24.4	III
CY26	954.1	black shale	1.46	20.73	Trace	77.23	2.04	−39.2	III
CY27	970.3	gray shale	1.44	41.46	Trace	57.94	0.6	−2.6	III
CY28	988.05	black shale	1.50	34.45	Trace	64.55	1	−15.0	III
CY31	999.2	black shale	1.39	35.63	Trace	62.97	1.4	−13.0	III
CY32	1019.3	black shale	1.53	39.73	Trace	59.97	0.3	−5.5	III
CY33	1035.1	black carbonaceous shale	1.52	42.87	Trace	56.16	0.97	−0.2	III
CY34	1035.3	black shale	1.48	34.86	Trace	63.04	2.1	−14.5	III
CY35	1048.7	black carbonaceous shale	1.46	38.35	Trace	60.85	0.8	−8.1	III
CY36	1050.8	gray-black shale	1.46	29.72	Trace	68.18	2.1	−23.5	III

a (%), b (%), c (%) and d (%) represent the volume percentages of sapropelinite, exinite, vitrinite and inertinite in shale maceral groups, respectively. Kerogen type index (KTI) = (100 × a + 50 × b − 75 × c − 100 × d)/100. The organic matter is predominantly type III (KTI < 0), the organic matter is predominantly type II2 (0 ≤ KTI < 40), the organic matter is predominantly type II1(40 ≤ KTI < 80), the organic matter is predominantly type I (KTI ≥ 80).

**Figure 2:**
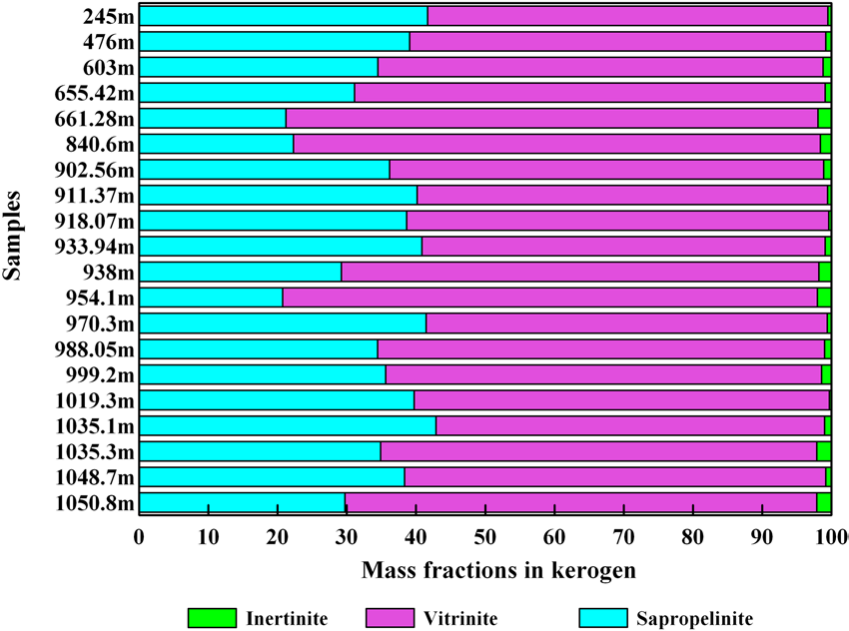
Bar plot of maceral composition showing the mass fractions of inertinite, vitrinite, exinite, and sapropelinite for all shale samples obtained from the Chaiye 2 well.

**Figure 3:**
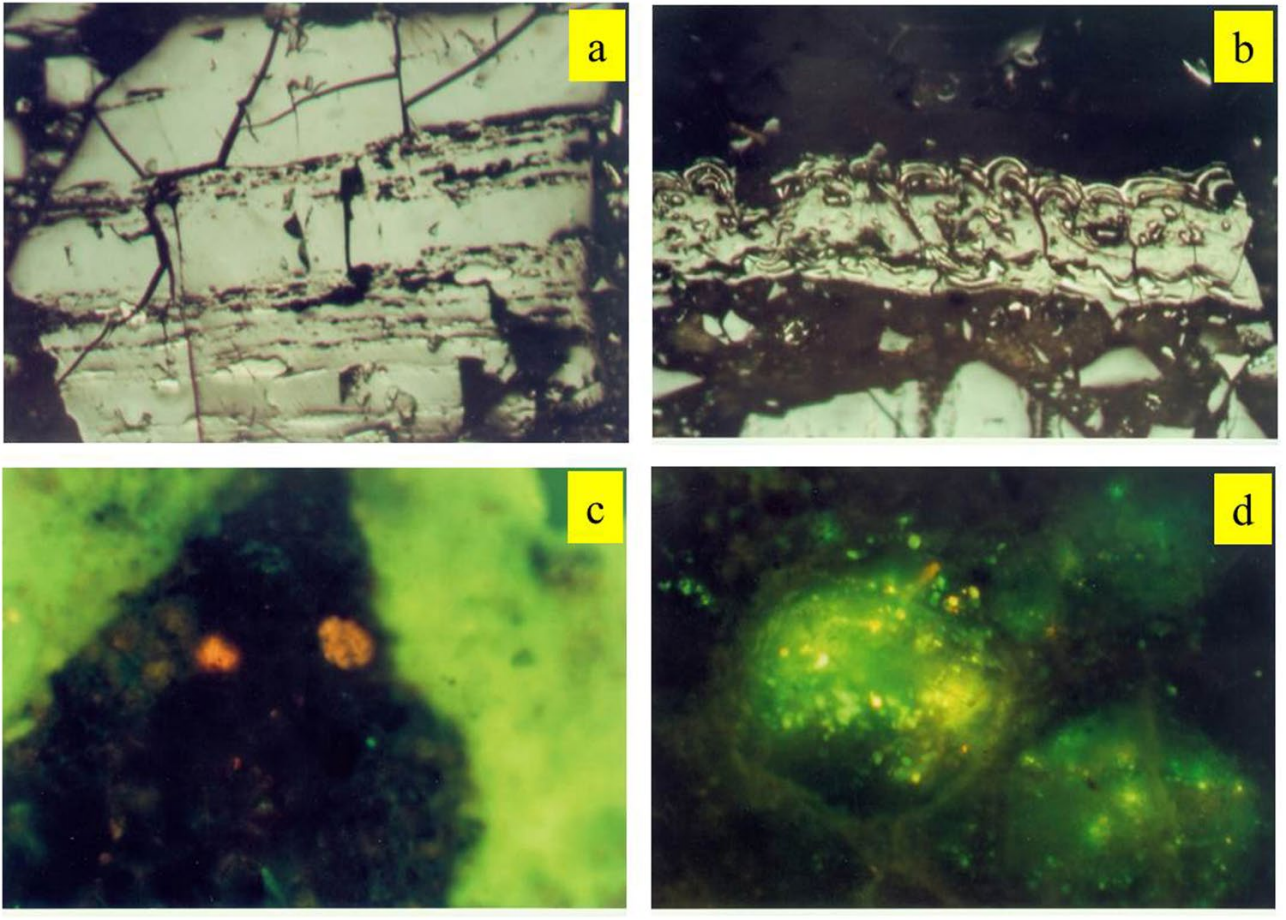
Photomicrographs of telocollinite (**a**), fusinite (**b**), mineral bituminous groundmass (**c**), and organic inclusions (**d**) observed in shale samples obtained from the Chaiye 2 well located in the Qaidam Basin under reflected light (**a**,**b**) and transmitted light (**c**,**d)** (field width = 0.2 mm).

The maceral contents of the shale samples are shown in Table 2 and in selected photomicrographs (Fig. 2). The shale samples contain high vitrinite contents (mean content of 64.22%) and trace amounts of exinite. These samples also contain abundant sapropelinite (20.73–42.87%) and small amounts of inertinite (mean content of 1.15%) (Table 2, Fig. 2). Telocollinite was the most common form of vitrinite (Fig. 3a), and the inertinite was mainly composed of fusinite (Fig. 3b). Mineralized bituminous groundmass was the main form of sapropelinite (Fig. 3c), and organic inclusions were identified in some samples (Fig. 3d).

## Molecular composition of hydrocarbons

### *n*-Alkanes and isoprenoid hydrocarbons

**Figure 4:**
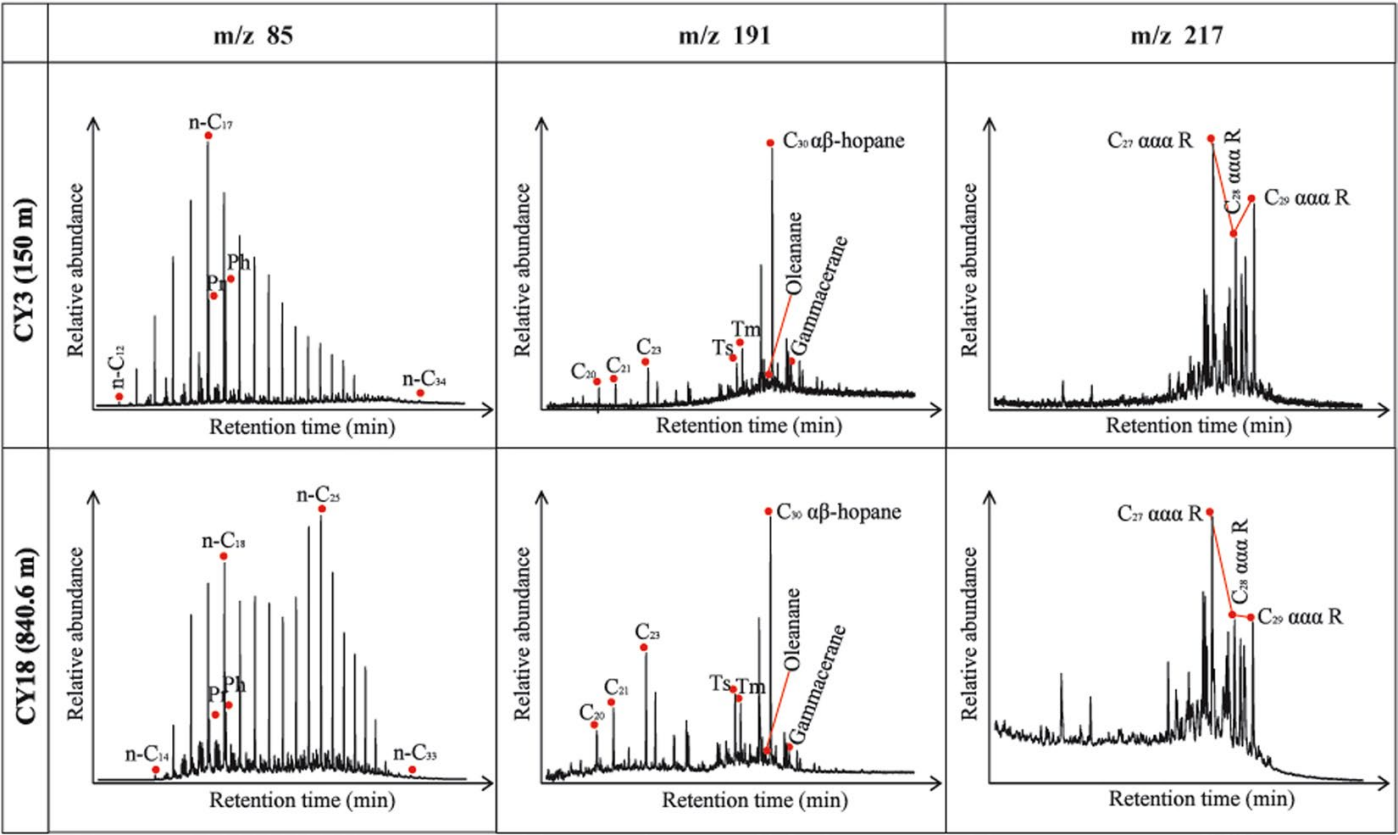
Ion chromatograms of m/z 85 (*n*-alkanes and isoprenoid hydrocarbons), 191 (tricyclic to pentacyclic terpanes) and 217 (steranes) for saturate hydrocarbon (saturate hydrocarbon were separated from by column chromatography). Within depth range of 40 to 661.28 meters, the CY3(150 m) is representative of all samples. Within dep th range of 840.6 to 1050.8 meters, the CY18(840.6 m) is representative of all samples.

The *n*-alkanes and isoprenoid hydrocarbons were distributed differently in samples obtained from different depths (Fig. 4, CY3(150 m)-m/z 85). The samples obtained from 40 to 661.28 meters contained *n*-alkanes ranging from *n* -C_12_ to *n*-C_34_. The *n* -alkane distributions were unimodal in these samples, and the samples display a consistent maximum at *n*-C_17_. The ranges of *n*-alkanes differ between the samples obtained from depths of 40 to 661.28 meters and those from 840.6 to 1050.8 meters (Fig. 4, CY18(840.6 m)-m/z 85). The *n* -alkane distribution pattern observed in the samples obtained from 840.6 to 1050.8 meters reflected abundant *n* -C_14_ to *n* -C_33_. Moreover, this pattern displayed a bimodal distribution with a consistent maximum at *n* -C_25_ and prominence at *n* -C_18_.

The studied samples had carbon preference index (CPI) values that range from 0.97 to 1.13 (Table 3). The $\sum n{{\rm{C}}}_{21}^{-}/\sum n{{\rm{C}}}_{22}^{+}$ ratios of the shale samples obtained from depths between 40 and 661.28 meters and 840.6 and 1050.8 meter ranged from 1.60 to 9.43 and 0.42 to 1.78, respectively (Table 3).

**Table 3:** Molecular marker parameters and biomarker indexes for extracts from shale samples obtained from the Chaiye 2 well located in the Qaidam Basin.

Sample	Pr/Ph	Pr/n-C_17_	Ph/n-C_18_	CPI	A	B	C_27_(%)	C_28_(%)	C_29_(%)	C	D	E	F	G	H	DBT/P	MPI	Rc(%)
CY1	0.78	0.58	0.77	1.02	1.64	1.36	33.26	27.40	39.34	0.43	0.47	0.08	0.01	0.58	0.45	0.19	0.67	1.04
CY2	0.87	0.70	0.84	1.05	2.23	1.41	32.21	24.83	42.96	0.41	0.44	0.08	0.02	0.60	0.51	0.19	0.61	1.00
CY3	0.85	0.39	0.58	1.08	4.83	1.02	41.84	26.36	31.80	0.39	0.42	0.11	0.03	0.57	0.42	0.23	0.81	1.12
CY4	1.04	0.42	0.44	1.04	4.11	0.98	41.17	26.38	32.45	0.35	0.40	0.08	0.02	0.59	0.47	0.16	0.85	1.15
CY5	0.72	0.59	0.77	1.00	5.38	1.37	32.89	25.97	41.14	0.39	0.44	0.08	0.02	0.56	0.50	0.18	0.82	1.13
CY6	0.85	0.52	0.68	1.05	5.19	1.30	34.22	26.72	39.06	0.43	0.45	0.08	0.02	0.57	0.49	0.12	0.93	1.20
CY7	0.85	0.52	0.68	1.05	5.19	1.13	36.57	25.07	38.35	0.48	0.56	0.10	0.08	0.59	0.56	0.11	0.97	1.22
CY8	0.93	0.29	0.34	1.13	2.49	1.25	34.89	27.20	37.91	0.43	0.51	0.10	0.05	0.61	0.49	0.09	0.94	1.20
CY9	1.01	0.43	0.47	0.99	5.01	0.87	44.30	22.88	32.82	0.46	0.45	0.11	0.05	0.61	0.45	0.16	1.04	1.26
CY10	1.63	0.47	0.30	1.01	2.10	0.84	44.85	24.83	30.32	0.46	0.48	0.11	0.03	0.54	0.59	0.14	1.10	1.30
CY11	1.1	0.20	0.19	1.03	4.89	0.91	44.29	22.76	32.95	0.47	0.45	0.11	0.03	0.60	0.57	0.12	1.10	1.30
CY12	1.85	0.44	0.20	1.02	1.60	0.89	43.00	22.61	34.38	0.46	0.49	0.09	0.04	0.59	0.57	0.16	1.04	1.26
CY13	1.34	0.50	0.39	1.00	1.81	0.97	42.93	25.51	31.56	0.46	0.47	0.11	0.04	0.61	0.55	0.17	1.06	1.27
CY14	1.59	0.27	0.19	1.01	3.38	1.02	40.08	23.71	36.20	0.42	0.42	0.12	0.03	0.58	0.53	0.12	1.17	1.34
CY15	1.36	0.08	0.06	1.03	3.17	1.00	38.35	26.48	35.17	0.43	0.48	0.11	0.05	0.52	0.51	0.14	1.16	1.34
CY16	1.19	0.09	0.10	1.09	9.43	1.05	37.62	26.40	35.98	0.43	0.46	0.11	0.04	0.51	0.47	0.56	1.19	1.35
CY17	1.48	0.35	0.25	1.01	1.86	1.14	36.78	27.03	36.19	0.49	0.50	0.11	0.04	0.56	0.50	0.11	1.21	1.37
CY18	1.48	0.35	0.38	1.01	1.01	0.70	46.90	25.95	27.15	0.45	0.43	0.07	0.07	0.59	0.54	0.28	1.24	1.39
CY19	1.4	0.38	0.35	1.03	1.07	0.71	47.30	24.74	27.97	0.51	0.42	0.10	0.07	0.59	0.57	0.09	1.40	1.48
CY20	1.35	0.28	0.32	1.06	0.42	0.62	48.07	27.12	24.81	0.45	0.38	0.09	0.03	0.58	0.55	0.07	1.19	1.35
CY21	1.27	0.33	0.37	1.00	0.75	0.94	43.14	25.39	31.48	0.48	0.44	0.09	0.02	0.60	0.55	0.07	1.26	1.40
CY22	1.05	0.29	0.34	1.04	1.78	0.94	41.98	26.74	31.28	0.38	0.39	0.09	0.04	0.58	0.56	0.12	1.31	1.43
CY23	0.85	0.32	0.39	0.97	0.90	0.84	43.00	24.43	32.57	0.47	0.42	0.11	0.04	0.58	0.55	0.09	1.28	1.41
CY24	0.94	0.38	0.44	1.03	0.89	0.63	50.98	24.98	24.04	0.46	0.41	0.09	0.05	0.61	0.62	0.09	1.18	1.35
CY25	1.15	0.35	0.36	0.97	1.26	0.65	46.83	27.09	26.08	0.50	0.43	0.09	0.02	0.58	0.54	0.10	1.36	1.46
CY26	1.09	0.34	0.45	1.08	0.51	0.72	47.21	25.92	26.87	0.47	0.40	0.07	0.03	0.59	0.54	0.11	1.18	1.35
CY27	1.08	0.41	0.44	0.99	0.72	0.89	42.41	26.82	30.77	0.42	0.39	0.12	0.04	0.58	0.51	0.10	1.09	1.29
CY28	1.01	0.40	0.48	0.99	1.37	0.77	45.80	25.04	29.15	0.50	0.41	0.08	0.05	0.59	0.53	0.13	1.28	1.41
CY29	1.14	0.44	0.45	1.04	0.93	0.73	48.49	24.93	26.59	0.44	0.39	0.10	0.11	0.58	0.62	0.14	1.11	1.31
CY30	1.14	0.36	0.39	1.04	1.11	0.68	47.92	25.22	26.87	0.45	0.40	0.10	0.03	0.58	0.61	0.14	1.42	1.49
CY31	0.97	0.35	0.44	1.06	1.01	0.61	52.47	24.02	23.51	0.45	0.40	0.10	0.04	0.56	0.63	0.12	1.27	1.40
CY32	1.06	0.40	0.52	1.06	1.32	0.73	47.91	24.95	27.15	0.47	0.40	0.08	0.04	0.57	0.57	0.09	1.17	1.34
CY33	1.23	0.40	0.47	1.04	1.70	0.82	45.11	26.06	28.83	0.47	0.41	0.11	0.03	0.59	0.58	0.17	1.34	1.45
CY34	1.19	0.39	0.44	1.02	1.05	0.79	46.99	23.92	29.09	0.52	0.43	0.10	0.04	0.56	0.53	0.12	1.19	1.36
CY35	1.14	0.33	0.43	1.06	0.82	0.83	44.63	25.00	30.37	0.43	0.39	0.11	0.03	0.58	0.60	0.15	1.23	1.38
CY36	0.94	0.34	0.43	1.07	0.95	0.90	42.18	26.22	31.60	0.43	0.39	0.11	0.04	0.58	0.60	0.10	1.21	1.37

Pr, Ph, *n*-C_17_, *n*-C_18_, Ts, Tm, DBT, P,MPI, MP and Rc represent pristine, phytane, *n*-heptadecane, *n*-octadecane, 18α (H) −22, 29, 30-trinorhopane, 17α (H) −22, 29, 30-trinorhopane, dibenzothiophene, phenanthrene, methylphenanthrene index, methylphenanthrene (MPI = 1.5(3-MP + 2-MP)/(P + 9-MP + l-MP)), equivalent vitrinite reflectance (Rc = 0.6MPI + 0.64), respectively. Carbon preference index (CPI) = (C_17_ + C_19_ + C_21_ + C_23_ + C_25_)/(C_16_ + C_18_ + C_20_ + C_22_ + C_24_) /2 + (C_17_ + C_19_ + C_21_ + C_23_ + C_25_)/(C_18_ + C_20_ + C_22_ + C_24_ + C_26_)/2. A represents the ratio of lower molecular weight (≤C_21_) *n*-alkanes ($\sum n{{\rm{C}}}_{21}^{-}$) to higher molecular weight (≥C_22_) *n*-alkanes ($\sum n{{\rm{C}}}_{22}^{+}$), B represents the ratio of C_29_ regular steranes to C_27_ regular steranes. C_27_(%), C_28_(%) and C_29_(%) represent the ratio of C_27_αα-20R, C_28_αα-20R and C_29_αα-20R to the total of C_27_, C_28_ and C_29_αα 20R sterane. C, D, E, F, and G represent the parameter of sterane C_29_αα-20S/(20S+20R), the parameter of sterane C_29_-ββ/(ββ+αα), gammacerane index(gammacerane/C_30_ αβhopane), oleanane index(oleanane/C_30_ αβ hopane), the parameter of hopane C_31_-22S/(22S + 22R), respectively. H represents the ratio of Ts to total of Ts and Tm.

Isoprenoid hydrocarbons were abundant in all of the shale samples and were represented by pristane (Pr) and phytane (Ph) (Fig. 4). The Pr/Ph ratios of the samples obtained from depths between 40 and 661.28 meters and 840.6 and 1050.8 meters range from 0.72 to 1.85 and 0.85 to 1.48, respectively (Table 3). The Pr/n-C_17_ and Ph/n-C_18_ values were very low and ranged from 0.08 to 0.70 and 0.06 to 0.84, respectively (Table 3).

### Tricyclic to pentacyclic terpanes

The tricyclic to pentacyclic terpanes identified by m/z 191 are shown in Fig. 4 -m/z 191. Additionally, the key parameters calculated for these tricyclic to pentacyclic terpanes are displayed in Table 3.

Tricyclic terpanes C_19_ to C_29_ were identified in the shale samples. The content of C_22_ and C_27_ were very low. The most prominent peak in the tricyclic terpanes series was that of C_23_, as shown in Fig. 4 -m/z 191. The tricyclic terpanes C_26_ to C_29_ were characterized by doublets in the Ion chromatograms, and the doublets contained S and R isomers. Moreover, pentacyclic terpanes from C_27_ to C_35_ were present. Of these, C_30_ (hopane) had the highest relative abundance, whereas the abundance of C_28_ was unusually low. Trisnorhopane (C_27_) and homohopanes (C_31_^+^) appeared as doublets (18α(H)and 17α(H); 22S and 22R epimers, respectively) in the Ion chromatograms for all of the studied shale samples (Fig. 4 -m/z191). Oleanane and gammacerane also appearen in the Ion chromatograms (Fig. 4 -m/z191).

The gammacerane index (gammacerane/C_30_αβhopane) values of the studied shale samples range from 0.07–0.12 (Table 3). The oleanane index (oleanane/C_30_αβhopane) values of these samples ranged from 0.01–0.11 (Table 3). The doublets of trisnorhopane (C_27_) contained 18α (H)-trisnorhopane (Ts) and 17α (H)-trisnorhopane (Tm) isomers. For these samples, the maturity index C_31_22S/(22S + 22R) and Ts/(Ts + Tm) values of the pentacyclic terpane series ranged from 0.51 to 0.61 and 0.42to 0.63, respectively (Table 3).

### Steranes

The mass chromatograms of m/z 217of the studied shale samples reflect enrichments in the 14α(H) and 17α(H) structural isomers, with a predominance of C_27_ or C_29_ relative to C_28_ regular steranes and diasteranes (C_27_–C_29_) and low abundances of short-chain pregnanes (C_20_ and C_21_) (Fig. 4 -m/z217).

For the studied shale samples, the ratios of C_29_ to C_27_ regular steranes (C_29_/C_27_ regular steranes) ranged from 0.61–1.41, with an average value of 0.92. The maturity indexes of the steranes were C_29_αα20S/(20S + 20R) and C_29_ββ/(ββ + αα), and the values of these indexes range from 0.35–0.52 and 0.38–0.56, respectively. Additionally, the percentage of the steranes C_27_αα20R, C_28_αα20R and C_29_αα20R ranged from 32.21–52.47%, 22.61–27.40%, and 23.51–42.96%, respectively (Table 3).

### Aromatics

**Figure 5:**
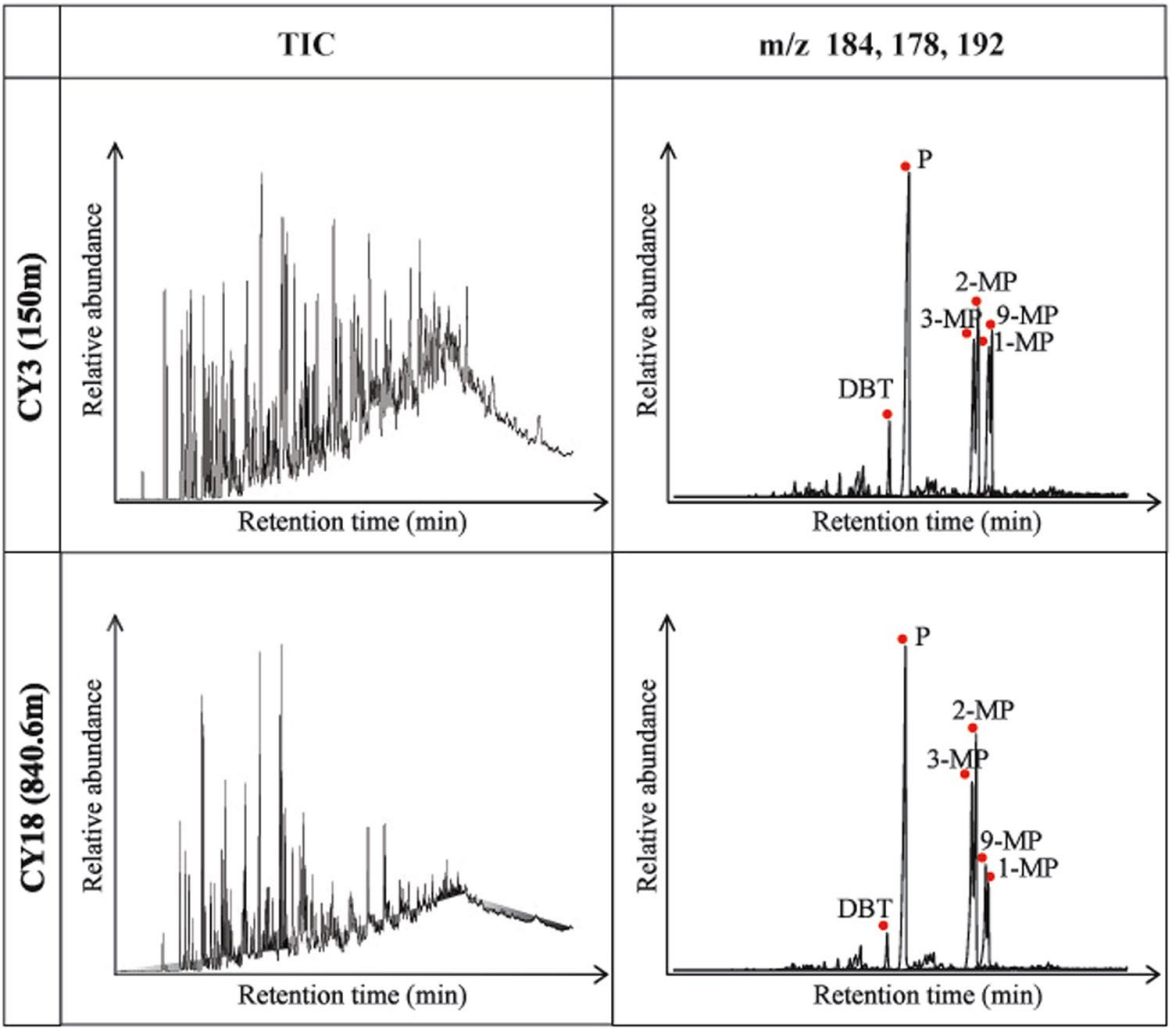
Gas chromatograms (TIC) and ion chromatograms of m/z 184 (dibenzothiophene), 178 (phenanthrene) and 192 (methylphenanthrenes) for aromatics of extracts from the studied samples. Within depth range of 40 to 661.28 meters, the CY3(150 m) is representative of all samples. Within depth range of 840.6 to 1050.8 meters, the CY18(840.6 m) is representative of all samples.

The total ion chromatograms (TIC) of the aromatic fractions of the studied shale samples are shown in Fig. 5. The major aromatics were naphthalene series compounds (methyl-, dimethyl-, and trimethylnaphthalenes), phenanthrene series compounds (methyl-, dimethyl-, and trimethylphenanthrenes), biphenyl series compounds (methyl- and dimethylbiphenyl), fluorene, dibenzofuran series compounds (methyl- and dimethyldibenzofuran), dibenzothiophene and methyldibenzothiophene, chrysene and methylchrysene, fluoranthene and pyrene series compounds (methyl- and dimethylfluoranthene and pyrene), perylene, and benzofluoranthene and benzopyrene, which were dominated by phenanthrene and methylphenanthrenes.

The methylphenanthrene index (MPI), which is based on phenanthrene and methylphenanthrenes (3-, 2-, 9-, and 1-) (Fig. 5 -m/z 184, 178, 192), was calculated as a maturation parameter^18^. The MPI values of the studied shale samples range from 0.61 to 1.42. The equivalent vitrinite reflectance (Rc), which is based on an empirical relationship between the MPI and vitrinite reflectance, was used to assess maturity^18^. The Rc (%) values of the studied shale samples range from 1.00% to 1.49% (Table 3).

The ratio of dibenzothiophene to phenanthrene (DBT/P) is thought to be an indicator of the depositional environment, origin and source rock lithology^19^. The values of the DBT/P ratio range from 0.07 to 0.56 (Table 3).

## Discussion

### Kerogen microscopy characteristics

The kerogen assemblage was recognized as dispersed organic matter, which includes phytoclasts (such as structureless amorphous organic matter, plant fragments, and marine-derived organic matter)^20^. The observation of kerogen with incident light and reflected light microscopy indicates that the organic matter found within the shale from the Chaiye 2 well is type III kerogen that contains a moderate amount of sapropelinite, traces of exinite, a high abundance of vitrinite and a low content of inertinite (Fig. 2, Table 2). The type of kerogen present can be described by the kerogen type index (KTI), which is calculated from the mass fractions of the different components of the kerogen^21^. According to the calculated results, the KTI values of all of the shale samples from the Chaiye 2 well were less than zero, reflecting type III kerogen^21^ (Table 2).

### Total organic carbon and types of organic matter

**Figure 6:**
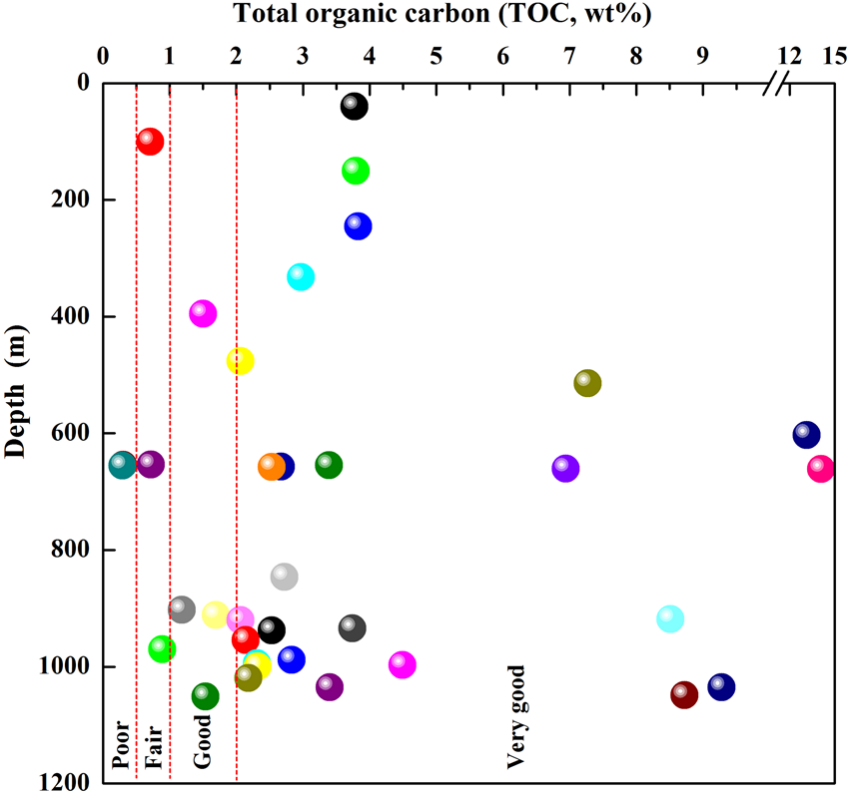
Distribution of total organic carbon (TOC, wt%) versus depth (m) for the shale samples obtained from the Chaiye 2 well.

TOC (wt%) was used to determine the abundance of organic matter and to evaluate the hydrocarbon generation potential of the samples^22^. Based on the TOC contents, 75% of the samples can be classified as “very good” source rock quality (>2.0%), 11% of the samples had “good” source rock quality (1.0–2.0%), and 8% of the samples had “poor to fair” source rock quality (0.5–1.0%). Only two samples (CY11 and CY13) displayed “poor” quality (<0.5%)^23^ (Table 1, Fig. 6).

**Figure 7:**
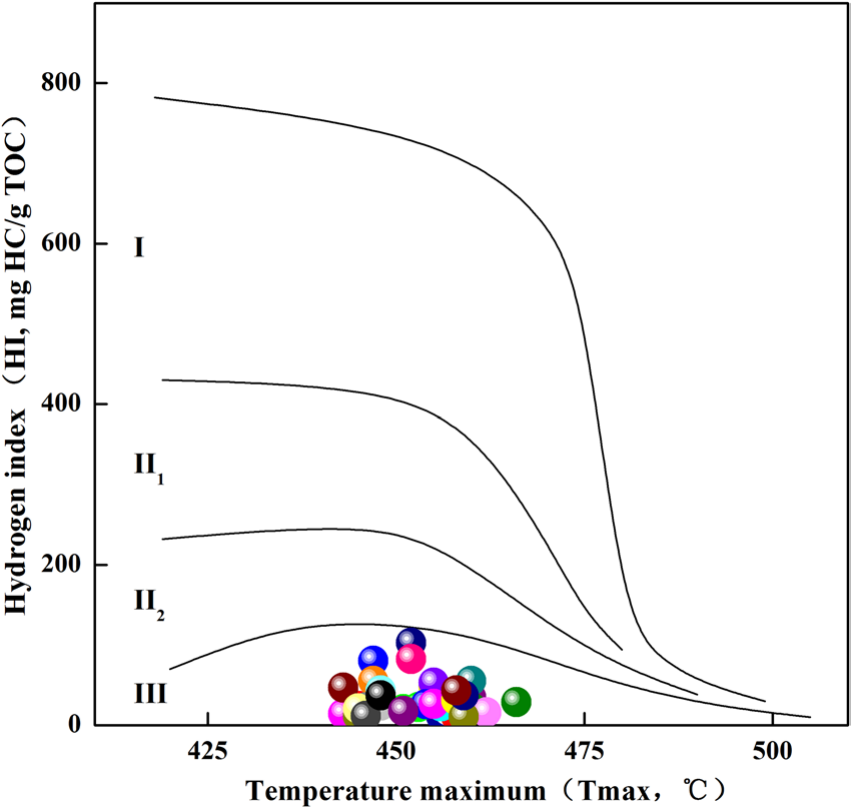
Plot of hydrogen index (HI) versus Tmax showing the kerogen types of the shale samples obtained from the Chaiye 2 well.

The shale samples obtained from the Chaiye 2 well had low HI values (Table 1). That range of Tmax values may have resulted from the low HI values, as the organic matter was predominantly type III (Fig. 7). The organic matter types of determined using Rock-Eval pyrolysis were consistent with the microscopic observations.

**Figure 8:**
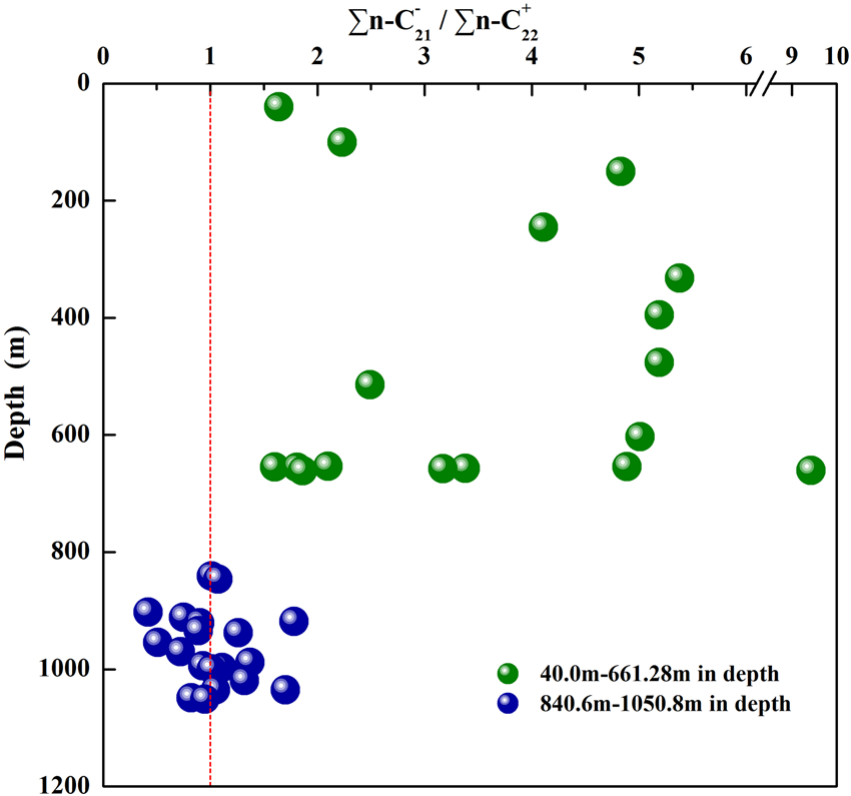
Distribution of *n*-C_21_^−^/*n*-C_22_^+^ versus depth for the shale samples obtained from the Chaiye 2 well.

**Figure 9:**
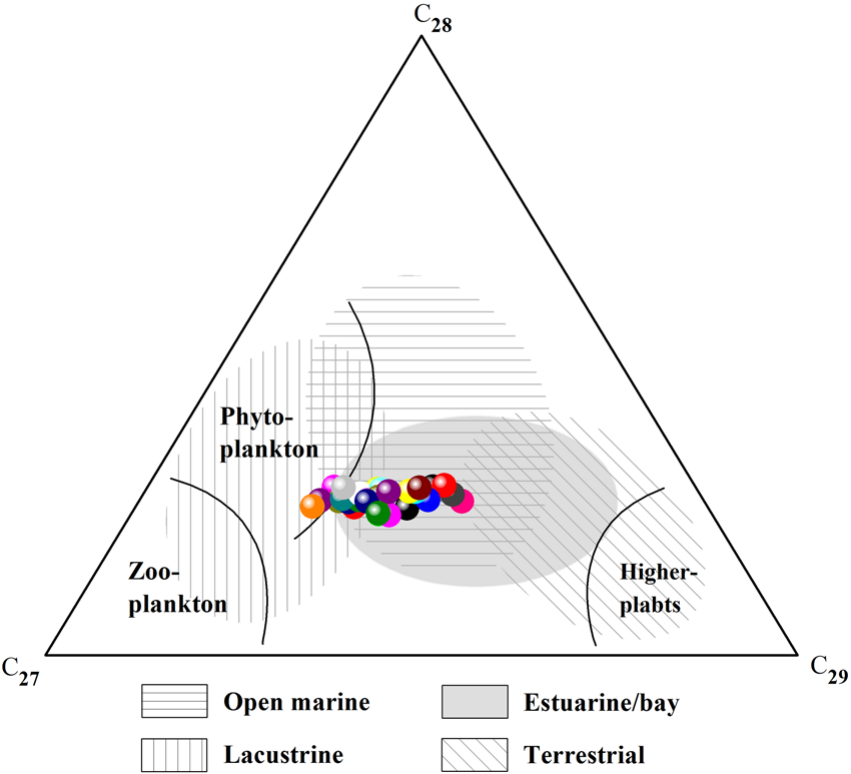
Ternary plot showing the relative contents of C_27_, C_28_ and C_29_ αα 20R steranes. Paleoenvironmental and source interpretations based on Armelle *et al*.^29^.

**Figure 10:**
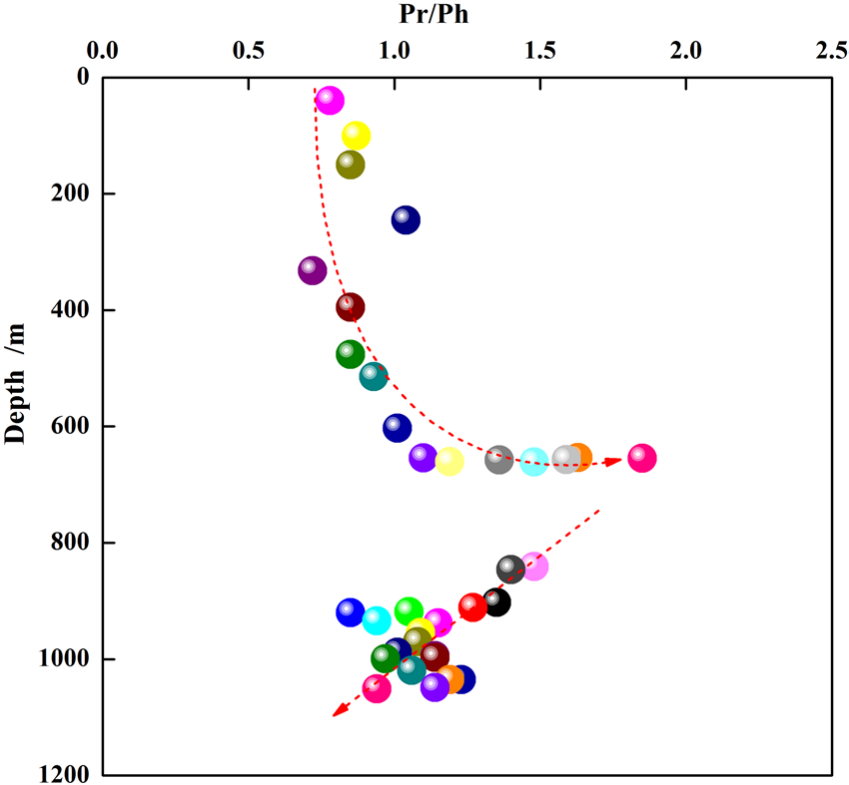
Ratio of pristane to phytane with depth in shale samples obtained from the Chaiye 2 well.

**Figure 11:**
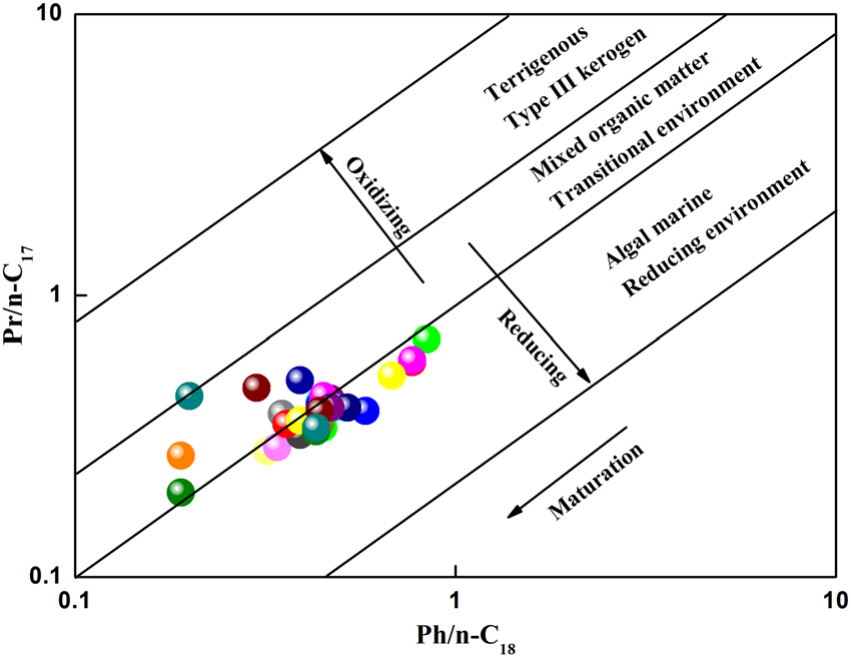
Plot of Pr/n-C17 versus Ph/n-C18 for shale samples obtained from the Chaiye 2 well. (interpretive scheme from Armelle *et al*.^29^).

**Figure 12:**
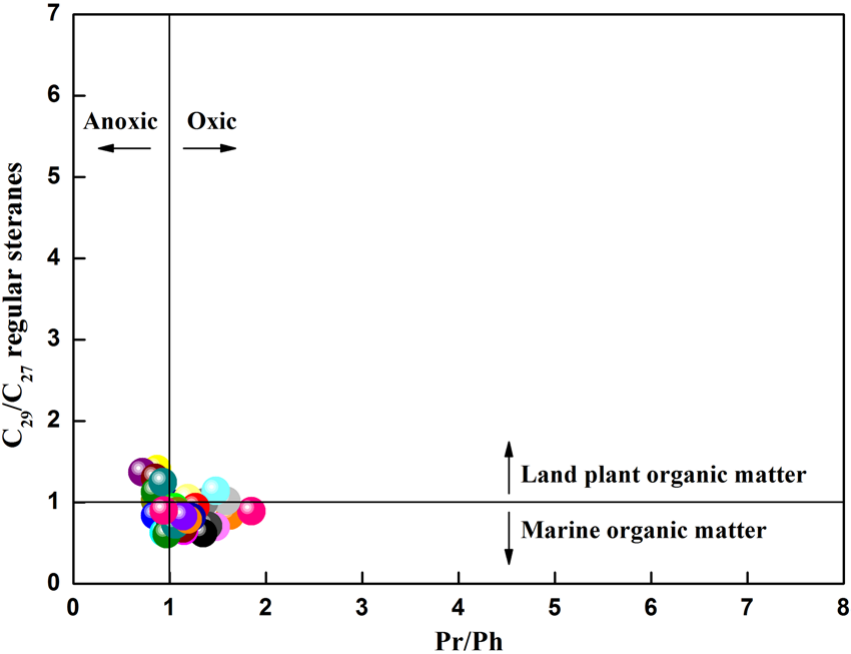
Depositional environment and organic matter input of the shale samples obtained from the Chaiye 2 well, as inferred from the ratio of Pr/Ph versus the ratio of regular sterane C_29_/C_27_.

**Figure 13:**
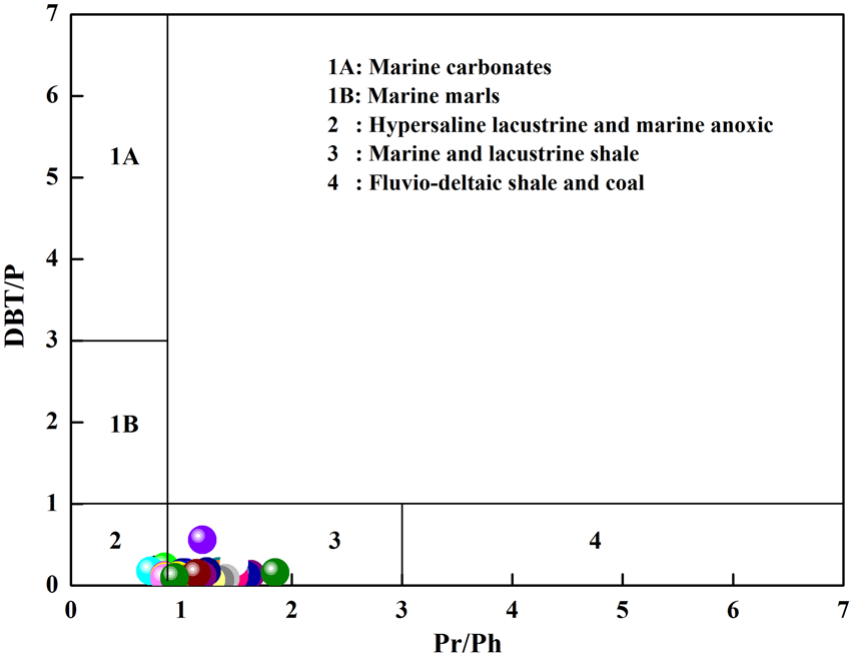
Ratio of dibenzothiophene to phenanthrene (DBT/P) plotted against the ratios of pristane to phytane (Pr/Ph) to determine shale depositional environments.

**Figure 14:**
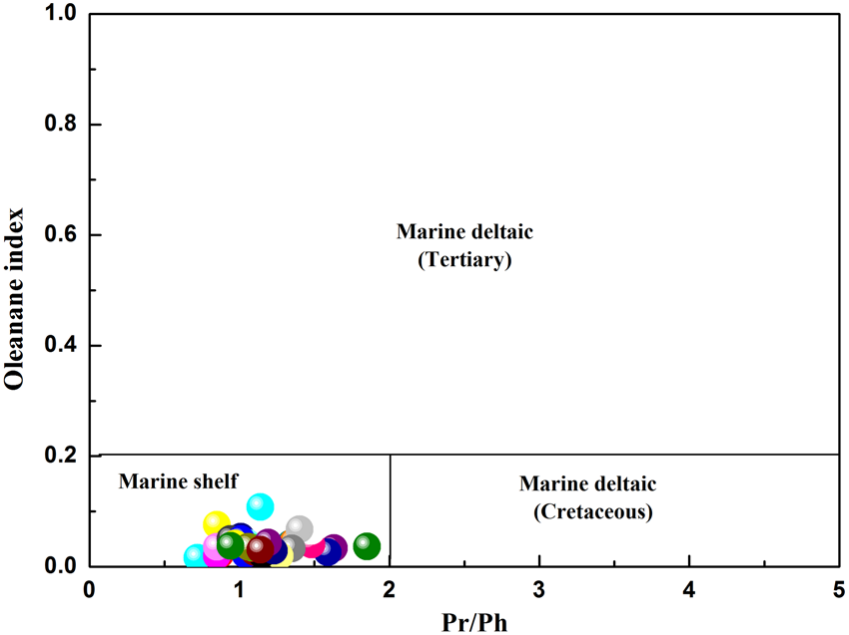
Plot of oleanane index values versus the pristane to phytane (Pr/Ph) ratio, indicating the depositional environment of the shale from the Chaiye 2 well.

**Figure 15:**
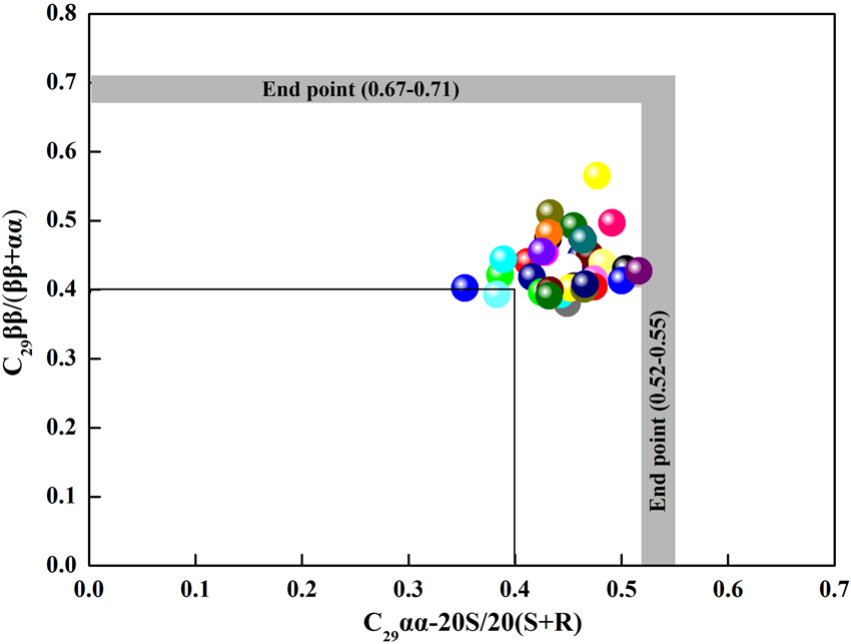
Plot of the sterane index C_29_ββ/(ββ + αα) versus the sterane index C_29_αα20S/(20S + 20R), indicating the maturity of the shale samples obtained from the Chaiye 2 well.

**Figure 16:**
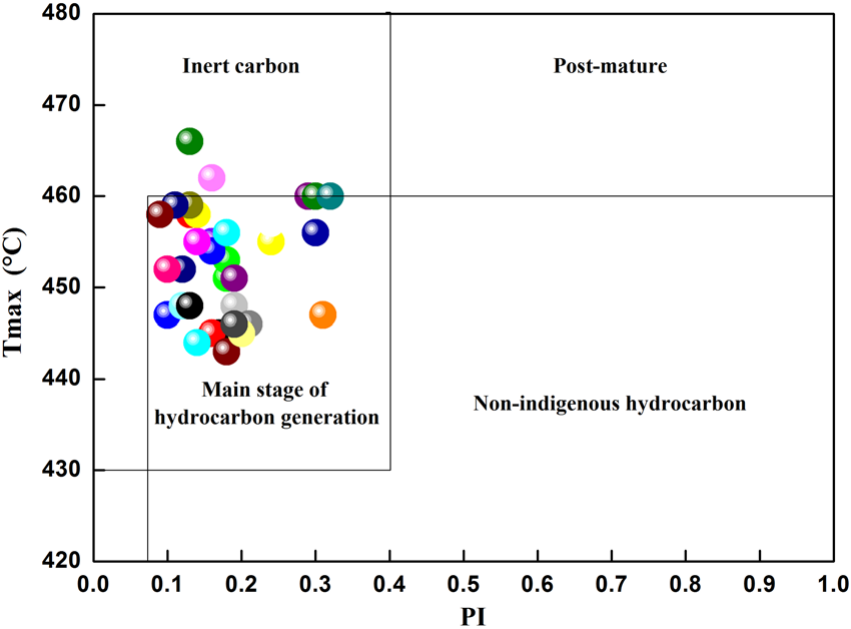
Plot of pyrolysis-based Tmax versus production index (PI) values reflecting the degree of maturation and nature of the hydrocarbon products in the shale samples obtained from the Chaiye 2 well in China.

### Sources of the hydrocarbons

The *n*-alkanes in shale from the Chaiye 2 well exhibit different distributions and compositional characteristics (Fig. 4 -m/z85), leading to change of some parameters. The studied samples had high $\sum {{\rm{nC}}}_{21}^{-}/\sum {{\rm{nC}}}_{22}^{+}$ values from 40 to 661.28 meters, with a low $\sum {{\rm{nC}}}_{21}^{-}/\sum {{\rm{nC}}}_{22}^{+}$ values from 840.6 to 1050.8 meters (Table 3, Fig. 8). These data suggest that the organic matter in the shale at depths of 40 to 661.28 meters was derived from aquatic organisms^24^ and at depths of 840.6 to 1050.8 meters was associated with a combination of aquatic organism and land plant sources^25^.

The relative amounts of C_27_–C_29_ αα 20R steranes can be used to indicate differences in the sources of organic matter^26^, because C_29_ and C_27_ sterols are derived primarily from terrestrial plants and zooplankton, respectively^27^. The contents of C_27_ ααα 20R steranes in the shale samples obtained from the Chaiye 2 well were similar to or slightly higher than the contents of C_29_ αα 20R steranes (Fig. 9), indicating that the shale is associated with a mix of terrestrial and marine organic sources^28^.

### Depositional environments

The depositional environment of the studied samples was extrapolated from the pristane/phytane (Pr/Ph), Pr/n-C_17_, Ph/n-C_18_, C_27_, C_28_ and C_29_ αα steranes, gammacerane and oleanane biomarker indexes.

The distributions of C_27_, C_28_ and C_29_ αα 20R steranes can be used to determine the depositional environments of shales^29^. Notably, the steranes of all of the samples plot in the estuarine/bay portion of the ternary diagram (Fig. 9).

The Pr/Ph ratio generally indicates the redox conditions of the depositional environment and varies with water depth and input type. Low Pr/Ph values reflect suboxic environments, deep water and marine inputs^19,30^. The Pr/Ph values of the samples found at depths of 40–661.28 meters in the Chaiye 2 well tended to increase with increasing sample depth (Fig. 10). By contrast, the Pr/Ph values in samples found at depths of 840.6–1050.80 meters tended to decrease with increasing sample depth (Fig. 10). Thus, the depositional environment of the studied samples transitioned from deep to shallow to deep, which is typical of sediments found in marine-continental transitional sedimentary facies. The relationship between the isoprenoid Pr/n-C_17_ and Ph/n-C_18_ ratios in the studied shale supports this interpretation and indicates a weakly oxic to weakly reducing pattern characteristic of sediments deposited in alternating sea and riverine facies^29^ (Fig. 11). This conclusion is also supported by the plot of the Pr/Ph ratio versus the C_29_/C_27_ regular sterane ratio^31^ (Fig. 12).

Gammacerane is an important C_30_ triterpane and may originate from tetrahymanols, which are widespread in marine sediments^32,33^. High gammacerane contents are typical of high-salinity environments and commonly result from hypersalinity and suboxidation at depth^34^. Therefore, gammacerane content can be used to identify the existence of stratified water columns in the depositional environments of marine and non-marine source rocks^35^. The shale samples obtained from the Chaiye 2 well had low the gammacerane index (gammacerane/αβC_30_ hopane) values. This range suggests weakly reducing, brackish conditions during the deposition of the source rocks^36^. The ratio of dibenzothiophene to phenanthrene (DBT/P) is thought to be an indicator of depositional environment, organic matter source and the source rock lithology^19^. The DBT/P values of samples from the Chaiye 2 well decreased with depth (Table 3). A plot of the DBT/P and Pr/Ph ratios (Fig. 13) shows that the samples obtained from the Chaiye 2 well fell on the boundary between marine and lacustrine shales and hypersaline lacustrine and anoxic marine deposits.

Oleanane, a biomarker indicative of higher terrigenous plants, has been suggested as an indicator of angiosperms (flowering plants)^37^. The ratio of oleanane to C_30_ hopane (deemed the oleanane index) provides information regarding depositional environments and source rock ages^38^. Oleanane index value greater than 0.2 indicate that the sample was deposited during the Tertiary and in a marine deltaic environment^19^. By contrast, oleanane index values less than 0.2 are characteristic of Cretaceous source rocks deposited in marine deltaic or marine shelf environments^19^. Figure 14 can be used to assess the depositional environment of the shale samples based on the Pr/Ph ratios and oleanane index values. The figure suggests that the studied shale samples are likely associated with a marine shelf depositional environment.

### Maturity

The maturities of the samples obtained from the Chaiye 2 well in the Qaidam Basin were studied using the Ro and Tmax values and several biomarker parameters.

The Ro (%) values of the samples were grester than 0.7% (Table 2), indicating a high degree of maturity. Based on the Ro values, 80% of the samples can be characterized as highly mature (1.3–2.0%), and 20% of the samples can be characterized as mature (0.7–1.3%)^18^ (Table 2).

The Tmax values of the samples were greater than 440 °C (Table 1), indicating mature to highly mature stages^39^.

The CPI values of the n-alkanes^30^, the sterane isomerization parameters C_29_αα20S/(20S + 20R) and C_29_ββ/(ββ + αα)^40^, the hopane parameters C_31_ααα22S/(22S + 22R) and Ts/(Ts + Tm)^40^, the methylphenanthrene index (MPI) and the equivalent vitrinite reflectance (Rc)^18^ are considered effective maturity indicators.

For the studied shale samples, the CPI values were close to 1.0 (Table 3) and indicative of the mature stage.

Most of the samples yielded high values (>0.4) of the sterane index C_29_ββ/(ββ + αα), except for samples CY20, CY22, CY27, CY29, and CY39. The end point of sterane index (C_29_αα20S/(20S + 20R) and C_29_ββ/(ββ + αα)) is 0.52–0.55 and 0.67–0.71, respectively^40^. And the sterane index greater than 0.4 indicate that the organic matter in samples is mature^40^. The values of both of these parameters indicate that the organic matter in the samples obtained from the Chaiye 2 well is mature^40^ (Fig. 15).

A mature state was inferred for the organic matter of all samples based on the values of the hopane index C_31_22S/(22S + 22R) and Ts/(Ts + Tm) (Table 3). The hopane indexes indicate that the samples obtained from the Chaiye 2 well are mature^40^.

The MPI and the equivalent vitrinite reflectance (Rc, %) are also considered to be highly effective maturity indicators^18^. The MPI and Rc values (Table 3) indicate that the samples obtained from the Chaiye 2 well are mature to highly mature^18^.

### Hydrocarbon-generating potential

The gas potential of shale can be evaluated using several standard methods. Zou (2010) and Zumberge (2010) suggested that shale should meet some geochemical criteria be associated a high shale gas potential^41,42^. For example, the TOC content should be greater than 2.0%, the Ro value should be higher than 0.8–1.1%, and the kerogen should be type II or III. In addition, Nie (2009) and Li (2011) argued that TOC contents greater than 1.0% effectively indicate areas of high shale gas potential in China^43,44^. For the shale samples obtained from the Chaiye 2 well, the TOC contents of 75% of the samples were greater than the 2% threshold value (average TOC content: 4.67%), and the TOC contents of 86% of the samples are greater than 1.0% (Table 1). All of the samples reflected a high maturity level that is adequate for the generation of gas. The kerogen type indicates a marine shelf depositional environment and the presence of type III organic matter. Hence, our data and interpretations, as well as the relatively high TOC contents and high maturities of the organic matter in the Chaiye 2 well indicate that the studied shale is suitable for commercial shale gas production.

Additionally, the Tmax and PI values obtained via the Rock-Eval pyrolysis analyses be used to assess the nature of the hydrocarbon products and the degree of maturation in the samples^45^. The relationship between Tmax and PI shows that the shale samples from the Chaiye 2 well in China are in the main stage of hydrocarbon generation (Fig. 16).

Therefore, the organic geochemical characteristics and petrographic results suggest that the shale samples obtained from the Chaiye 2 well have very good gas generation potential.

## Methods

### Organic geochemical analyses

The shale samples were analyzed using GC-MS and Rock-Eval pyrolysis. The TOC contents of the samples were also determined.

The 36 shale samples were crushed and ground to mesh size smaller than 100, and subjected to Rock-Eval pyrolysis to determine their TOC and volatile hydrocarbon (HC) (S_1_), remaining HC generation potential (S_2_), production index (PI = S_1_/[S_1_+S_2_]), hydrogen index (HI = S_2_/TOC × 100), and Tmax value (the temperature corresponding to the maximum value of S_2_).

The Rock-Eval pyrolysis analyses were conducted using a Rock-Eval 6 instrument made in France. The Rock-Eval pyrolysis and TOC analyses were performed on 130 mg of ground material from the shale samples. The ground sample material was heated to 850 °C at a rate of 10 °C/min in a helium atmosphere.

Approximately 150 g of ground material from each shale sample was subjected to Soxhlet extraction using chloroform for 72 h at a constant temperature of 70 °C. The extracts from the shale samples were deasphalted by precipitation with n-hexane and filtration. The deasphalted maltenes were fractionated into saturates and aromatics via column chromatography using activated silica gel and aluminum oxide (v:v = 3:1) with n-hexane and methylene chloride, respectively^46^. Saturates and aromatics were then analyzed using GC-MS.

The GC-MS analyses were performed using an Agilent 6890N GC interfaced with a 5973 MS. An Agilent HP-5 column (30 m × 0.25 mm i.d., 0.25 μm film thickness) was used. The injection temperature was 70 °C (2 min hold), and the temperature program was 4 °C/min from 80 °C to 290 °C (30 min hold). The flow rate of the carrier gas (He) was 1.1 mL/min, and the pressure was 2.4 kPa. The sample injection volume was 1.0 L, and the split ratio was 10:1. Electron impact (EI) ionization at 70 eV was used for the ion source. The temperatures of the transfer line and ion source were 280 °C and 230 °C, respectively. The parent ion was m/z 285, the activating voltage was 1.5 V, and the scanning range was from m/z 35 to 600.

### Petrographic analyses

The vitrinite reflectance values were measured in random mode and reported in Ro (%). The samples were mounted in resin and were then ground into pellets and polished using alumina–ethanol slurry. The measurements were performed under oil immersion at a wavelength of 546 mm using a Leitz Orthoplan/MPV-SP photometer microscope system.

Petrographic analyses were performed on the polished shale samples under reflected white light following conventional methods using the Leitz Orthoplan/MPV-SP photometer microscope system. Each sample was measured at least 500 times.

## Conclusions

The geochemical and petrographic analyses of the Carboniferous shale penetrated by the Chaiye 2 well in the Qaidam Basin suggest the following conclusions. The organic matter within these rocks originated in a marine shelf depositional environment. This conclusion is supported by various geochemical parameters, such as Pr/Ph; Pr/n-C_17_, Ph/n-C_18_, C_27_, C_28_ and C_29_ αα steranes, C_29_/C_27_ regular steranes, the gammacerane index, the oleanane index, and the DBT/P ratio of the aromatics. The high TOC contents of these rocks include large amounts of vitrinite and sapropelinite, and this organic matter is characterized as a highly mature type III kerogen. These conclusions are supported by the values of various geochemical parameters, such as TOC, KTI, HI, Ro, Tmax, the CPI of the n-alkanes, the sterane parameters C_29_αα20S/(20S + 20R) and C_29_ββ/(ββ + αα), the hopane parameters C_31_22S/(22S + 22R) and Ts/(Ts + Tm), the MPI and the Rc of the aromatics as well as the molecular composition of the hydrocarbons. The Carboniferous shale penetrated by the Chaiye 2 well has very good gas generation potential, as indicated by the large amounts of highly mature type III organic matter in the shale (the TOC contents of 86% of the samples exceeded 1.0%), which originated in a marine shelf depositional environment.

## Electronic supplementary material

supplementary information
