# Supplementary material for: The origin, type and hydrocarbon generation potential of organic matter in a marine-continental transitional facies shale succession (Qaidam Basin, China)
Source: Sci Rep. 2018 Apr 26;8:6568. doi: 10.1038/s41598-018-25051-1 (PMC5920063; doi:10.1038/s41598-018-25051-1)
Supplement: Supplementary file 1 — supplementary information [file 41598_2018_25051_MOESM1_ESM.pdf]

The origin, type and hydrocarbon generation potential of organic matter in a marine-continental transitional facies shale succession (Qaidam Basin, China)

Guo-Cang Wang[1], Min-Zhuo Sun[1,*], Shu-Fang Gao[2,+] , Li Tang[2,+]

[1]Key Laboratory of Petroleum Resources, Gansu Province/ Key Laboratory of Petroleum Resources Research, Institute of Geology and Geophysics/ The Analytical Service center, Research Center of Oil and Gas Resources, Northwest Institute of Eco-environment and Resources, CAS, Lanzhou 730000, PR China.

[2]PetroChina Qinhai Oilfield Research Institute of Exploration ＆ Development，Dunhuang 736202,PR China.

[*]Corresponding.author@email: sunmz04@sina.com

[+]These authors contributed equally to this work

Supplementary information:

1. Page 2, Line 26

Geological background

2. Page 4, Line 7-12

To investigate the origin, type and hydrocarbon generation potential of shale, we studied the characteristic of its insoluble organic matter by Rock-Eval to determine the Total Organic Carbon content(TOC), free hydrocarbons (S1), hydrocarbon generative potential (S2), temperature (Tmax) at themaximum of the S2 peak, and production index (PI=S1/(S1+S2)), hydrogen index (HI=S2/TOC ×100).

3. Page 5, Line 1-5

Main geochemical characteristics of pyrolysis study on shale samples, as determined by Rock-Eval analysis. Total Organic Carbon (TOC) is given in percent. S1 represents

free hydrocarbons, S2 represents the hydrocarbon generative potential, Tmax represents the temperature at themaximum of the S2 peak. Production index (PI=S1/[S1+S2]) and hydrogen index (HI=S2/TOC×100).

4. Page 6, Line 1-7

Maceral groups, the vitrinite reflectance values (Ro) and proximate analysis of the studied shale samples. a (%), b (%), c (%) and d (%) represent the volume percentages of sapropelinite, exinite, vitrinite and inertinite in shale maceral groups, respectively. Kerogen type index (KTI) = (100×a+ 50×b - 75×c - 100×d)/100. The organic matter is predominantly type III (KTI<0), the organic matter is predominantly type II2 (0⩽KTI<40), the organic matter is predominantly type II1(40⩽KTI<80), the organic matter is predominantly type I (KTI⩾80).

5. Page 9, Line 1-17

Molecular marker parameters and biomarker indexes for extracts from shale samples obtained from the Chaiye 2 well located in the Qaidam Basin. Pr , Ph, *n*-C_{17}， *n*-C_{18}，Ts, Tm, DBT, P,MPI, MP and Rc represent pristine, phytane, *n*-heptadecane, *n*-octadecane, 18α (H) -22, 29, 30-trinorhopane, 17α (H) -22, 29, 30-trinorhopane, dibenzothiophene, phenanthrene, methylphenanthrene index, methylphenanthrene (MPI =1.5(3-MP+2-MP)/(P+9-MP+l-MP)), equivalent vitrinite reflectance (Rc=0.6MPI+0.64), respectively. Carbon preference index (CPI) = $(C_{17}+C_{19}+C_{21}+C_{23}+C_{25})/(C_{16}+C_{18}+C_{20}+C_{22}+C_{24})/2+(C_{17}+C_{19}+C_{21}+C_{23}+C_{25})/(C_{18}+C_{20}+C_{22}+C_{24}+C_{26})/2$. A represents the ratio of lower molecular weight(≤C_{21}) *n*-alkanes ($\sum nC_{21}^{-}$) to higher molecular weight (≥C_{22}) *n*-alkanes($\sum nC_{22}^{+}$)**,** B represents the ratio of C_{29} regular steranes to C_{27} regular steranes. C_{27}(%), C_{28}(%) and C_{29}(%) represent the ratio of C_{27}αα-20R, C_{28}αα-20R and C_{29}αα-20R to the total of C_{27}, C_{28} and C_{29}αα 20R sterane. C, D, E, F, and G represent the parameter of sterane C_{29}αα-20S/ (20S+20R), the parameter of sterane C_{29}-ββ/(ββ+αα), gammacerane index(gammacerane/C_{30} αβhopane), oleanane index(oleanane/C_{30} αβ hopane), the parameter of hopane C_{31}-22S/(22S+22R), respectively. H represents the ratio of Ts to

total of Ts and Tm.”

6. Page 13, Line 10-13

leading to change of some parameters. The studied samples had high $\sum nC_{21}^{-}/\sum nC_{22}^{+}$ values from 40 to 661.28 meters, with a low $\sum nC_{21}^{-}/\sum nC_{22}^{+}$ values from 840.6 to 1050.8 meters (Table 3, Fig. 8).

7. Page 17, Line 10-12

The shale samples obtained from the Chaiye 2 well had low the gammacerane index (gammacerane/$\alpha\beta C_{30}$ hopane) values.

8. Page 19, Line 6

were grester than 0.7%

9. Page 19, Line 10

were greater than 440 ℃

10. Page 19, Line 18

>0.4
